# The Role of Nootropic Supplements in Sports: Enhancing Cognitive and Physical Performance Simultaneously

**DOI:** 10.1002/fsn3.71819

**Published:** 2026-04-30

**Authors:** Zheng Yi

**Affiliations:** ^1^ Department of Physical Education Xi’an University of Finance and Economics Xi’an Shaanxi China

**Keywords:** brain–muscle axis, cognitive enhancement, neurotransmitter modulation, nootropic supplements, sports performance

## Abstract

Athletic competition in contemporary sports requires athletes to demonstrate physical fitness and their ability to think clearly while making choices and maintaining focus, and their mental abilities to work through weariness. Athletes use nootropic supplements, which include natural herbal compounds, synthetic racetams, cholinergic agents, adaptogens, and nutrient‐based formulations, as they seek to improve their cognitive abilities and physical performance. The review examines the nootropic effects of substances used in sports through their impact on neurotransmission, neuroendocrine control, energy production, synaptic plasticity, and neurovascular connections. The four key neurotransmitters dopamine, acetylcholine, serotonin, and GABA function to control motivation, attention, fatigue perception, and psychomotor control, while the creatine–phosphate shuttle and mitochondrial AMPK/PGC‐1α signaling pathways serve to maintain ATP levels in both the brain and muscles. The research explains specific exercise mechanisms that help different types of athletes perform better. Endurance athletes achieve performance improvements through greater central drive and nitric oxide‐mediated blood circulation. Strength athletes gain advantages through enhanced motor unit activation and energy storage capabilities. Team and combat athletes enhance their performance through better reaction times and increased capacity to handle pressure. Precision‐based athletes improve their performance through cholinergic focus, GABA balance, and motor skill development. The review demonstrates that nootropic supplements can serve as a performance enhancement strategy to improve cognitive and physical abilities when athletes use them in a safe manner, which matches their personal requirements.

## Graphical abstract

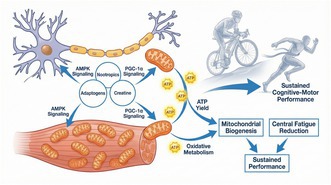

## 1 Introduction

The demands of modern competitive sports require athletes to develop multiple cognitive skills, which need to be assessed through more advanced measurement methods than basic strength, speed, and endurance tests. The ability of athletes to quickly understand their surroundings and handle information while performing precise movements under extreme physical and mental pressure determines their success in high‐performance environments. Across all sports disciplines, which include team sports, combat sports, endurance events, and precision‐based activities, decision‐making speed and accuracy, together with sustained attention, reaction time, working memory, and emotional regulation skills, have become the essential factors that determine competitive success (Prats Lara 2020; Medrano et al. 2022; Ribeiro and Poínhos 2023; Gibson et al. 2020).

Elite athletes must make quick decisions because they compete in environments that keep changing. Players in football, basketball, and hockey must perform continuous tactical updates while they create opponent movement forecasts and execute coordinated plays with their teammates during periods of intense physical activity. Combat sports require athletes to make quick threat assessments while they decide between defense and offense, and they must keep their attention on their opponents during physical attacks and mental stress. Endurance sports performance depends on cognitive processes because athletes need to manage their pacing, effort, and motivation throughout extended competitions, and when they face thermal, metabolic, or psychological challenges (Vine et al. 2025; Kennedy 2019; Patel et al. 2016; Caldenhove et al. 2018; Malík and Tlustoš 2022; Schifano et al. 2025; Smith et al. 2020; Van Cutsem and Pattyn 2024).

Nootropics have entered sports nutrition as an innovative solution to improve athletic performance because researchers recognize that optimal performance requires understanding both physical body functions and brain activity. Pre‐workout formulas, energy products, and recovery supplements now include caffeine, L‐theanine, creatine, tyrosine, citicoline, and multiple adaptogenic herbs because these ingredients help users stay alert and boost their energy levels while needing to enhance their focus, reaction speed, motivation, and mental endurance (Brand 2016; Paiva et al. 2024; Razazan et al. 2025; Lorca et al. 2023).

The review aims to present an all‐encompassing scientific assessment of current nootropic research on athletic performance. The review will achieve its objectives by first establishing the main nootropic categories that have relevance to sports and then studying their effects on human brain functions and body systems while assessing their impact on essential mental skills and physical performance, and finally examining safety standards, ethical issues, and regulatory requirements.

## 2 Methodology

The author conducted a thorough narrative review, which aimed to extract current scientific evidence. The author assessed how well these supplements improved mental functions that included attention, memory, and reaction time and physical abilities that included endurance, strength, and agility.

## 3 Classification of Nootropic Supplements in Sports

### 3.1 Natural (Herbal and Nutraceutical) Nootropics

Natural nootropics derived from plant sources and traditional medical systems represent one of the most widely used categories in sports nutrition. The compounds exist as multimodal substances that create four distinct effects by controlling stress responses, producing antioxidant and anti‐inflammatory benefits, increasing brain blood circulation, and managing monoaminergic neurotransmitter systems (Jędrejko et al. 2023). The substances function as adaptogens, which help athletes who deal with intense training, mental pressure, and persistent exhaustion. *Rhodiola rosea* functions as an established adaptogen that helps people reduce their mental and physical fatigue while extending their endurance and improving their cognitive skills during stress. The bioactive components of the plant, which include rosavins and salidroside, are believed to affect dopamine and serotonin pathways while they decrease excessive cortisol production through their impact on the hypothalamic–pituitary–adrenal (HPA) axis (Prats Lara 2020). These effects support psychological resilience, sustained motivation, and central fatigue resistance. The main medical application of *Ginkgo biloba* involves its ability to enhance blood flow in the brain and provide protection to neurons (Maqbool et al. 2025; Leonard 2024; H‐DAF et al., n.d.; Malík and Tlustoš 2023; Salum et al. 2024) (Table 1).

**TABLE 1:** Types of nootropics and mechanisms.

Nootropic	Primary mechanism	Cognitive effect	Physical effect	References
*Rhodiola rosea*	HPA axis modulation, cortisol reduction	Improves attention, mental resilience	Enhances endurance, delays fatigue	(Malík and Tlustoš 2023)
*Ginkgo biloba*	Increased cerebral blood flow	Improves memory and focus	Supports coordination and reaction time	(Maqbool et al. 2025)
*Panax ginseng*	Dopaminergic activation	Enhances alertness and working memory	Improves stamina and endurance	(Schifano et al. 2025; Hedayati‐Moghadam et al. 2025)
*Bacopa monnieri*	BDNF upregulation, antioxidant activity	Enhances learning and memory	Supports motor learning	(Schifano et al. 2025)
Gotu kola	Neuroprotective, anti‐inflammatory	Improves processing speed	Supports balance and coordination	(Maqbool et al. 2025)
Piracetam	AMPA receptor modulation	Improves memory and cognitive flexibility	Enhances psychomotor efficiency	(McEwen 2024)
Aniracetam	AMPA receptor facilitation	Improves verbal memory and focus	Reduces mental fatigue	(Jędrejko et al. 2023)
Oxiracetam	Neurotransmission enhancement	Improves attention and learning	Supports motor coordination	(Maan et al. 2025)
Noopept	Neuroplasticity, BDNF stimulation	Improves memory consolidation	Maintains cognitive performance under fatigue	(Leonard 2024)
Phenylpiracetam	Dopaminergic and noradrenergic stimulation	Increases alertness and vigilance	Enhances strength and endurance	(Frazer et al. 2025)
Alpha‐GPC	Acetylcholine precursor	Enhances focus and working memory	Improves neuromuscular output	(Gurjar et al., n.d.)
Citicoline (CDP‐choline)	Phospholipid synthesis, acetylcholine support	Improves attention and learning	Enhances psychomotor coordination	(Bell et al. 2025)
Huperzine A	Acetylcholinesterase inhibition	Improves cognitive processing	Enhances neuromuscular transmission	(Mancuso 2024)
Ashwagandha	HPA axis regulation	Reduces anxiety, improves focus	Supports strength and recovery	(Malík and Tlustoš 2023)
Cordyceps	Mitochondrial energy support	Improves alertness	Enhances aerobic endurance	(Onaolapo and Onaolapo 2024)
Caffeine	Adenosine receptor antagonism	Increases vigilance	Improves endurance and power	(Kang and Kim 2023)
Creatine	Phosphocreatine energy buffering	Improves memory and attention	Enhances high‐intensity performance	(Schifano et al. 2025)
Tyrosine	Catecholamine precursor	Maintains focus during stress	Supports motivation and endurance	(Lorca et al. 2023)
Theanine	GABAergic modulation	Improves attention, reduces anxiety	Stabilizes motor control	(Fioravanti et al. 2023)
Taurine	Neuromodulator, antioxidant	Enhances mental clarity	Supports cardiovascular endurance	(Ludyga et al. 2022)
Omega‐3 fatty acids	Anti‐inflammatory neuroprotection	Improves cognitive processing	Supports neuromuscular recovery	(Yongtawee et al. 2022)
B‐vitamins	Neurotransmitter cofactor	Enhances memory and attention	Supports energy metabolism	(Kalén et al. 2021)
Modafinil	Dopaminergic activation	Sustains alertness	Reduces perceived fatigue	(Fatima and Khare 2020)
Adrafinil	Wakefulness‐promoting mechanism	Improves vigilance and decision making	Supports cognitive–physical tasks	(Fioravanti et al. 2023)
Schisandra	Adaptogenic HPA regulation	Improves concentration	Supports endurance performance	(Solmaz and Erbaş 2023)
*Mucuna pruriens*	Dopamine precursor (L‐DOPA)	Enhances motivation and focus	Improves neuromuscular function	(Joshi Pranav 2013)
Pramiracetam	Cholinergic potentiation	Enhances learning and memory	Supports cognitive‐motor performance	(Pastina and Stewart 2025)
L‐carnitine	Mitochondrial fatty‐acid transport	Reduces cognitive fatigue	Enhances aerobic endurance	(Jędrejko et al. 2023)

### 3.2 Synthetic Nootropics (Racetams and Analogues)

Researchers developed synthetic nootropics, which included racetams and related compounds for medical treatment of neurological and cognitive disorders, but people now use these substances to improve their performance and cognitive abilities (Jędrejko et al. 2023). The compounds produced by this method show specific effects on glutamatergic and cholinergic systems while they also affect synaptic plasticity and cortical excitability (Salum et al. 2024). Piracetam serves as the primary example of racetam drugs, which scientists believe increase neuronal membrane fluidity while affecting both AMPA and NMDA receptor function to promote synaptic transmission and neuroplasticity (Gaurava et al. 2020). The substance has shown the ability to enhance cognitive processing and learning abilities, although research about its effects on athletes remains limited. Aniracetam and Oxiracetam function as two racetam compounds that provide different effects of anxiety reduction and stimulation, respectively (Pokrywka et al. 2025).

### 3.3 Cholinergic Nootropics

**FIGURE 1:**
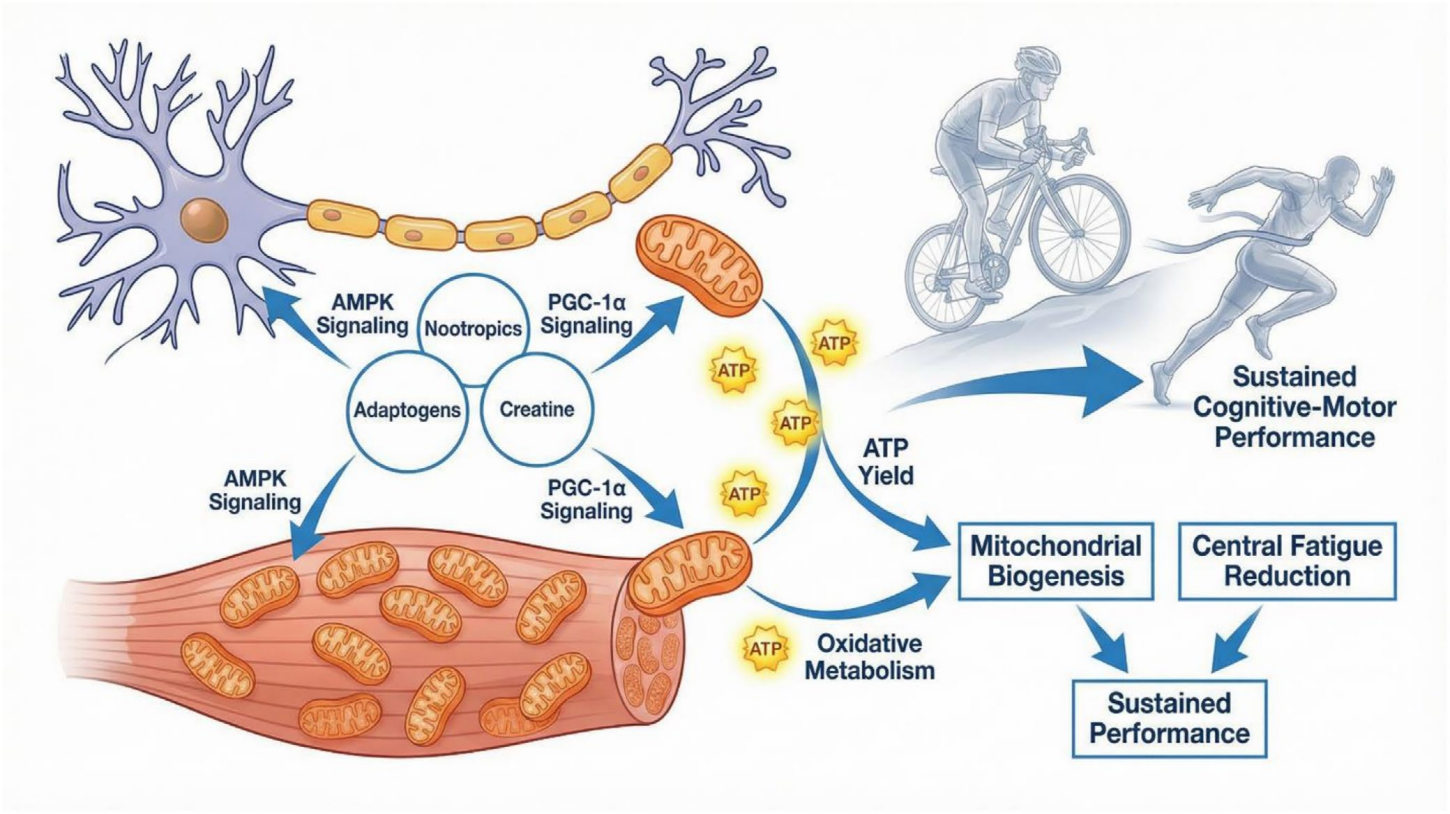
Mitochondrial Enhancement and AMPK/PGC‐1α Signaling.

Cholinergic nootropics target the acetylcholine system, which plays a central role in attention, learning, memory, and neuromuscular transmission (Pastina and Stewart 2025). The agents provide essential support for athletic performance because acetylcholine functions in both cognitive tasks and muscle movement control. Alpha‐GPC (α‐glycerylphosphorylcholine) and Citicoline (CDP‐choline) serve as bioavailable choline donors, supporting acetylcholine synthesis in the brain and at the neuromuscular junction (Prats Lara 2020). Huperzine A functions as a reversible acetylcholinesterase inhibitor, which increases acetylcholine levels at synaptic sites through its action of stopping acetylcholine degradation. The mechanism has the potential to improve cognitive abilities and enhance attention skills, but using it for extended periods creates hazards to health (Jędrejko et al. 2023) (Figure 1).

### 3.4 Adaptogens and Metabolic Enhancers

The category of adaptogens and metabolic enhancers exists as a separate functional category that helps people deal with stress while fighting fatigue and maintaining their metabolic functions (Prats Lara 2020). This group shares some characteristics with natural nootropics, but it primarily focuses on how the body handles stress while maintaining its energy levels. *Rhodiola rosea* , Ashwagandha ( *Withania somnifera* ), and Cordyceps serve as the main examples of this category (Jędrejko et al. 2023). Ashwagandha leads to cortisol level decreases, together with improvements in stress resilience and possible strength and aerobic capacity advancements (Malík and Tlustoš 2022). The compounds work to improve cognitive abilities because they lower physical stress levels while providing additional energy resources (Joshi Pranav 2013).

### 3.5 Nutrient‐Based and Amino Acid Nootropics

The most valid evidence backing sports nutrition exists through the nutrient‐based and amino acid nootropic category. These compounds function as essential components that support both neural pathways and muscle systems, which manufacturers use in their performance supplements.

Caffeine stands as the most researched nootropic‐ergogenic supplement, which boosts alertness and reaction speed, vigilance, and endurance performance through adenosine receptor blocking and increased catecholamine production (Fatima and Khare 2020).

The intake of creatine results in increased phosphocreatine buffering capacity within muscle tissues and brain tissues, which leads to improved strength and power, repeated sprint capacity, and cognitive abilities during fatigue and sleep deprivation. Tyrosine functions as a dopamine and noradrenaline synthesis precursor, which helps people maintain their cognitive abilities when facing stress and fatigue. Theanine functions as a caffeine companion, which helps users achieve relaxed alertness while decreasing their anxiety and jitteriness. Taurine, B‐vitamins, and Omega‐3 fatty acids protect neurotransmission and membrane stability and energy production, which enhances both mental performance and athletic ability (Fatima and Khare 2020).

## 4 Cognitive Domains Relevant to Sports Performance

Athletic performance depends on two core components, which allow athletes to concentrate on relevant task elements while blocking out all external disturbances. Athletes need to develop their ability to maintain focus through various sensory challenges because competitive situations involve both intense crowd sounds and quick changes in visual and audio information. Athletes during training and competition need to use three types of attentional processes, which include selective attention to relevant information, sustained attention through time, and divided attention for multiple streams of information (Kalén et al. 2021). The frontoparietal networks handle neurobiological mechanisms of attentional control while cholinergic and noradrenergic neurotransmission systems control its effects. Acetylcholine enhances sensory differentiation and attention by boosting signal strength that passes through cortical networks, whereas noradrenaline controls both arousal levels and vigilance states (Scharfen and Memmert 2019; Li et al. 2022).

Working memory enables people to store temporary information while they work toward their goals. Sports rely on working memory to enable athletes to develop tactical plans, understand their surroundings, and combine current game information with their past training (Aguiñaga et al. 2022). Athletes need to track their adversaries' present locations and upcoming moves and current game conditions, which requires them to do so multiple times each second. Athletes need to continuously update their current situation to maintain awareness of their environment while they execute their complex physical movements (Sato et al. 2022).

The measurement of reaction time and processing speed through testing procedures enables an accurate assessment of cognitive abilities, which impact athletic performance. The constructs measure how fast a person processes sensory information before they execute their physical response to it (Fanelli et al. 2022). Competitive sports use millisecond timing because it can decide who wins when athletes sprint, start, play ball sports, compete in combat, and execute defensive moves. The brain processes information at a higher speed when people perceive environmental cues, which leads to faster decision‐making about their motor responses (Yongtawee et al. 2022; Piskin et al. 2022; Trecroci et al. 2022; Xu et al. 2024; Rad et al. 2022; Guchait and Muggleton 2024; Spytska 2024; Cheng 2024; Abdoli et al. 2025) (Table 2).

**TABLE 2:** Cognitive domains and nootropic effects in sports.

Cognitive domain	Definition	Nootropic influence	Example supplement	Sports relevance	References
Attention & Selective Focus	Sustained focus and filtering relevant stimuli	Alpha‐GPC and citicoline improve attentional stability	Alpha‐GPC, Citicoline	Archery, shooting, basketball	(Pastina and Stewart 2025; Le Bars 2003)
Working Memory & Recall	Temporary storage and retrieval of information	Bacopa enhances memory consolidation and recall	*Bacopa monnieri*	Team sports, tactical games	(Medrano et al. 2022; Belcaro et al. 2015)
Reaction Time & Alertness	Speed of response to stimuli	Caffeine increases neural excitability and vigilance	Caffeine	Sprinting, football, martial arts	(Lorca et al. 2023; Burns et al. 2006)
Decision‐Making & Executive Function	Rapid evaluation and strategic planning	Modafinil improves cognitive flexibility and decision efficiency	Modafinil	Soccer, basketball, strategy sports	(Asadi et al. 2020; Canter and Ernst 2007)
Motor Learning & Skill Acquisition	Learning and refining movement patterns	Noopept enhances synaptic plasticity and learning	Noopept	Gymnastics, precision sports	(Al‐Karawi et al. 2016)
Cognitive Flexibility & Task Switching	Ability to shift attention between tasks	Racetams and aniracetam enhance adaptability	Piracetam, Aniracetam	Fencing, multi‐role sports	(Bakhtiari et al. 2017; Camfield et al. 2014)
Stress Resilience & Tolerance	Maintaining cognition under pressure	Ashwagandha reduces cortisol and anxiety	Ashwagandha	Martial arts, competitive sports	(Ball et al. 2011; Chengappa et al. 2013)
Focus Under Fatigue & Mental Endurance	Sustaining cognition during exertion	Tyrosine and creatine support neurotransmitters and energy	Tyrosine, Creatine	Endurance cycling, distance running	(Attia et al. 2012; Canter and Ernst 2003)
Memory Consolidation & Learning	Storing learned skills and experiences	Phenylpiracetam improves memory retention	Phenylpiracetam	Skill‐based sports	(Banerjee et al. 2021)
Vigilance & Wakefulness	Sustained alertness during prolonged tasks	Modafinil maintains wakefulness and focus	Modafinil	Endurance and tactical sports	(Barry et al. 2007)
Visual Processing & Spatial Awareness	Interpreting visual and spatial information	Ginkgo improves cerebral blood flow and processing	*Ginkgo biloba*	Ball sports, racquet sports	(Maqbool et al. 2025)
Response Control & Error Detection	Inhibiting incorrect actions and recognizing mistakes	Theanine and noopept stabilize cortical activity	Theanine, Noopept	Precision shooting, combat sports	(Bell and Williams 2019; Bell et al. 2015)
Perceived Exertion & Cognitive Fatigue	Mental perception of fatigue during effort	Tryptophan and bacopa reduce fatigue perception	Tryptophan, *Bacopa monnieri*	Endurance competitions	(Benson et al. 2019; Chan et al. 2007)
Motivation & Drive	Psychological drive to sustain performance	Phenylpiracetam enhances dopaminergic activity	Phenylpiracetam	Weightlifting, CrossFit	(Brimson et al. 2021)
Situational Awareness & Anticipation	Understanding dynamic environments and predicting actions	Rhodiola improves alertness and reaction readiness	*Rhodiola rosea*	Team sports, combat sports	(Malík and Tlustoš 2023)
Cognitive Load & Prioritization	Processing multiple stimuli efficiently	Tyrosine and citicoline support attentional control	Tyrosine, Citicoline	Strategy‐intensive sports	(Calabrese et al. 2008; Carrillo and Benitez 2000)
Focus Recovery	Regaining concentration after disruption	*Panax ginseng* supports sustained concentration	*Panax ginseng*	Tennis, boxing	(Schifano et al. 2025; Bell and Williams 2019)

## 5 Integrated Brain–Muscle Performance Framework

Athletic performance has become recognized as a result of brain and muscle systems, which work together with others in opposition to traditional beliefs that base athletic performance purely on muscular and metabolic functions (Chauhan 2025). The central nervous system (CNS) serves as the primary system that controls all aspects of movement execution, including force production, pacing strategies, and fatigue perception, since it combines cognitive, emotional, and physiological information. The integrated brain‐muscle performance framework shows that cognitive functions such as attention, motivation, and decision‐making processes serve as essential parts that determine how brain activity transforms into physical performance (Ó'Reilly and Delis 2022).

The brain–muscle axis establishes a two‐way communication system that connects brain areas to both spinal motor neurons and skeletal muscle fibers. The motor and premotor cortices produce motor commands that use corticospinal pathways to reach spinal motor neurons that activate muscle fibers for force and movement creation. The central nervous system receives continuous updates about force production, muscle length, and joint position through sensory feedback, which travels through afferent pathways from muscles, tendons, and joints (Qi et al. 2024). The system uses sensory data from multiple sources to create motor programs that improve movement precision. The axis functions because cognitive and emotional states really do impact it at different system levels. The prefrontal and limbic brain regions control motor cortical activity, which results in changes to motor unit recruitment and coordination patterns (Wu et al. 2024). The muscle activation process is affected by three factors, which include motivation, perceived effort, and emotional arousal. In high‐performance sport, where small changes in neural efficiency can have large effects on outcome, the functional integrity of the brain–muscle axis becomes a critical determinant of performance consistency and resilience under stress (Przewłócka et al. 2024).

Traditional fatigue and performance models use peripheral limitations as their primary focus, which includes muscle glycogen depletion, metabolite accumulation, and impaired excitation–contraction coupling (Ó'Reilly and Delis 2022). Current evidence shows that both factors contribute to fatigue, but central mechanisms play a major role in restricting performance. Central fatigue describes a gradual decrease in voluntary muscle control, which results from changes in cortical activity and neurotransmitter levels and central motor command functions. Central limitations lead to decreased motor unit recruitment, reduced firing frequency, and disrupted synchronization, which results in lower force output and movement accuracy, despite intact peripheral muscle strength (Van Cutsem and Pattyn 2024; Marocolo et al. 2023; Tedeschi 2023; Forelli et al. 2025; Jayasinghe 2025; Patel et al. 2024).

## 6 Neurotransmitter‐Mediated Mechanisms of Action

### 6.1 Enhanced Cholinergic Transmission

The neurotransmitter acetylcholine (ACh) plays a vital role in human cognitive functions, which include attention, learning, and memory, and in controlling motor activities. Cholinergic signaling in the central nervous system boosts cortical arousal and sensory processing and attentional focus through its ability to enhance signal‐to‐noise ratios in brain circuits (Davis et al. 2023). Acetylcholine functions as the main neurotransmitter in the peripheral nervous system because it activates muscle fiber depolarization, which leads to contraction at the neuromuscular junction. The cholinergic system functions as a fundamental pathway through which cognitive control interacts with physical performance (Yuan et al. 2025).

**FIGURE 2:**
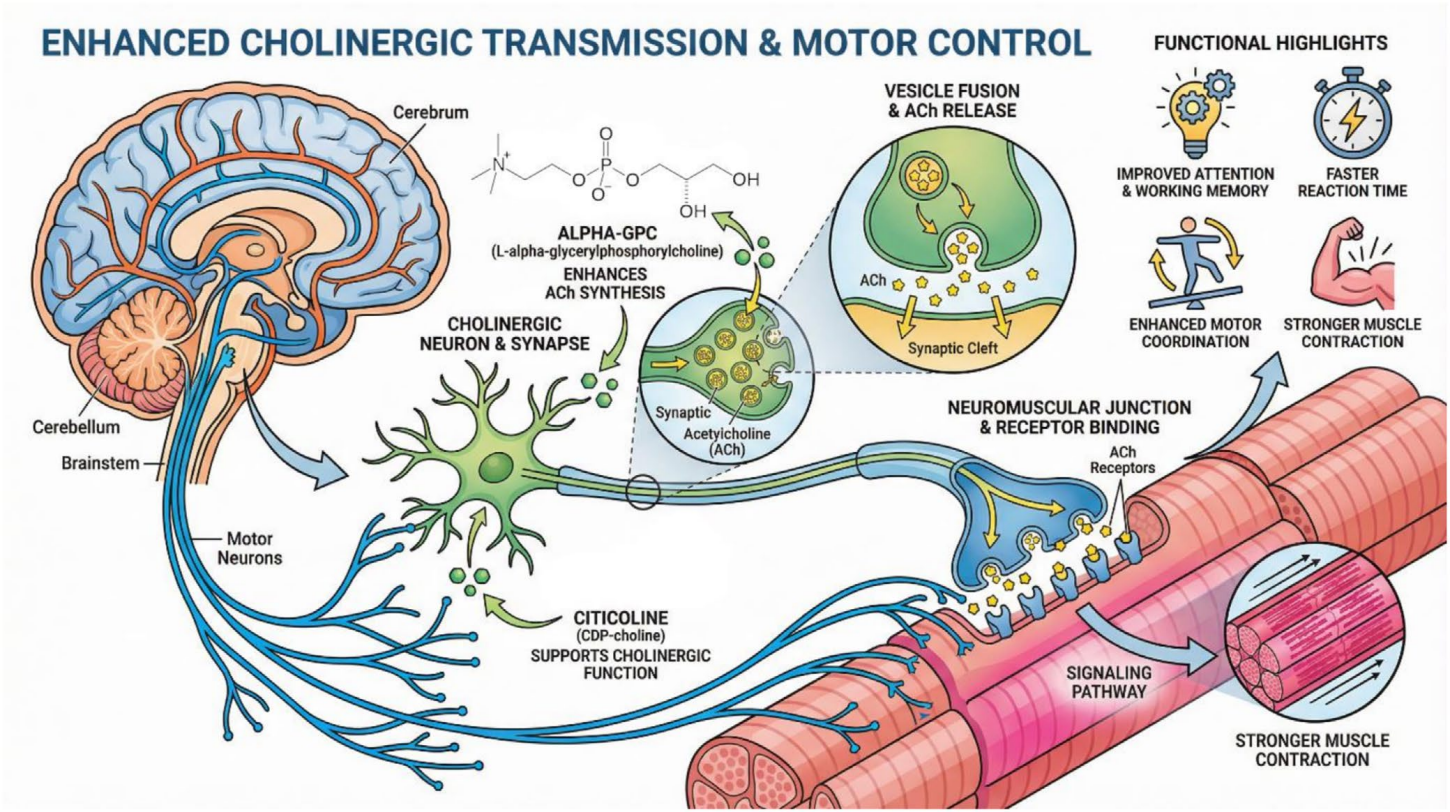
Enhanced cholinergic transmission and motor control.

The brain's cholinergic system enables people to focus their attention on tasks while maintaining their visual perception throughout sports activities, which require them to track moving objects and execute precise motor movements. The body uses acetylcholine to control muscle movement because its signaling system enables muscles to contract quickly and move together, which improves users' ability to control their body movements. The body experiences performance declines when people experience declines in cholinergic function that result from either fatigue, stress, or extended periods of cognitive work (Pascuzzi et al. 2026; Xu et al. 2025) (Figure 2).

### 6.2 Dopaminergic and Noradrenergic Activation

The catecholamine neurotransmitters dopamine and noradrenaline function as essential components that control human arousal and motivation, as well as executive function and motor control. Dopaminergic pathways connect essential functions, which include reward processing and goal‐directed behavior, together with motor drive regulation. Dopamine impacts an athlete's drive to work hard, their ability to maintain effort during exhaustion, and how they perceive their performance achievements. The decrease in dopaminergic signaling causes people to experience higher effort levels while their motivation decreases, which leads to central fatigue and performance deterioration (Hiraga et al. 2024).

Noradrenaline functions as a neurotransmitter that induces arousal and maintains vigilance while allowing people to change their focus of attention. The drug improves sensory processing in both sensory and cortical brain areas and enables quick changes to new environmental requirements. Optimal noradrenergic tone enables people to remain alert while their brains select responses, but both high and low norepinephrine levels will disrupt cognitive control and make people more sensitive to distractions and anxious feelings (Ferré et al. 2022; Kobayashi et al. 2022).

### 6.3 Serotonergic Regulation

The mood state, emotional control, and fatigue assessment of individuals are controlled by serotonin, which is also known as 5‐hydroxytryptamine or 5‐HT. People who exercise for extended periods or at high intensity will experience increased effort and fatigue and impaired physical performance when their central serotonin levels rise. The serotonin hypothesis of fatigue proposes that exercise‐induced fatigue leads to decreased performance because elevated serotonin signals in the brain change how people perceive their physical effort and motivation levels (Salvan et al. 2023).

**FIGURE 3:**
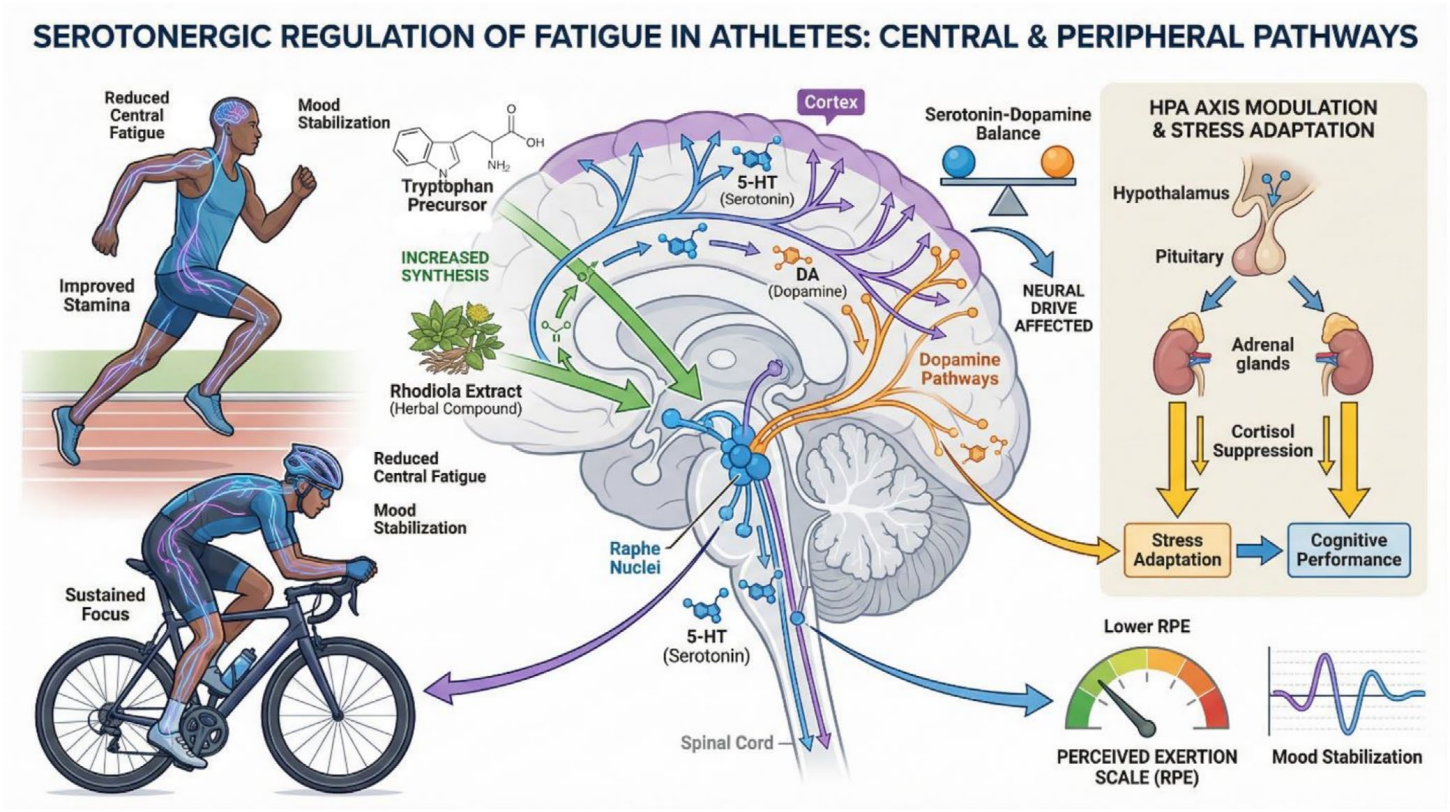
Serotonergic regulation of fatigue.

Serotonin functions as a vital component that helps athletes maintain stable moods while controlling their emotions to achieve psychological health and stress management. The body experiences mood disorders and anxiety, together with a decreased ability to handle stress because of disrupted serotonergic pathways, which lead to negative effects on athletic and training performance (Tian et al. 2023; Chen et al. 2022) (Figure 3).

### 6.4 GABAergic Modulation

**FIGURE 4:**
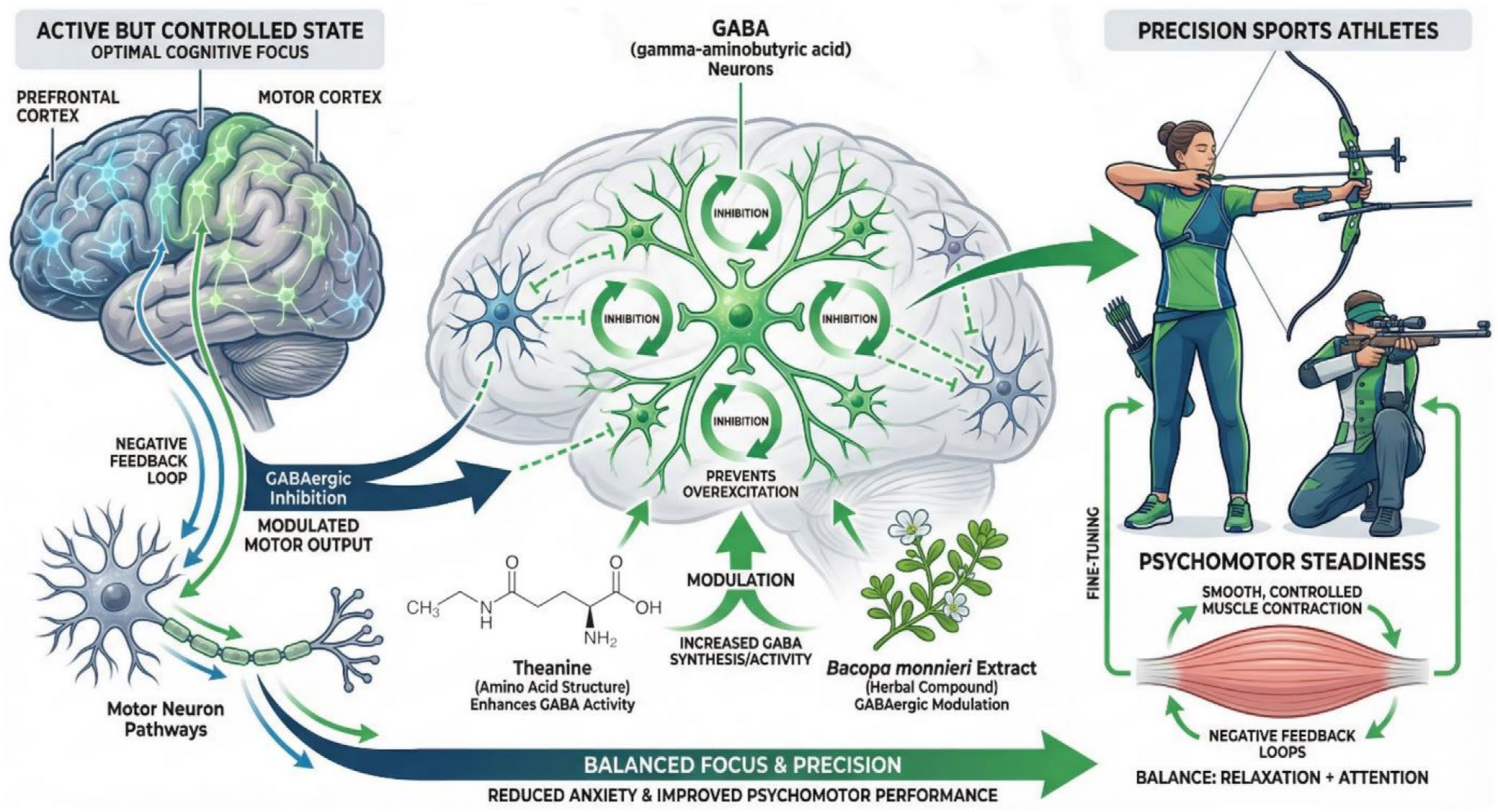
GABAergic modulation and focus balance.

Gamma‐aminobutyric acid (GABA) serves as the primary inhibitory neurotransmitter in the central nervous system, which utilizes GABA to regulate both emotional responses and stress reactions and neural function. GABAergic signaling enables cortical regions to maintain their optimal balance between excitation and inhibition, which allows individuals to sustain their focus, manage their emotional states, and execute precise movements. The athletic performance of an athlete depends on how their neural system handles excessive neural activity, which creates anxiety and jitters and disrupts their ability to move accurately. At the same time, their body system deals with excessive neural activity, which causes their body to become sluggish and lose mental sharpness (Ruenkoed et al. 2023). The optimal GABAergic modulation process enables people to maintain calm focus while decreasing their performance anxiety and achieving steady psychomotor control during competitive situations that require high performance. The particular situation holds crucial importance for sports that require athletes to perform exact movements because athletes must control their motor functions and their emotional states to achieve their highest performance level. Nootropic and nutraceutical compounds that influence GABAergic pathways may help regulate anxiety and enhance focus and balance, which supports a mental state that allows people to perform at their best (Koh et al. 2023) (Figure 4).

## 7 Mental Fatigue and Central Fatigue in Sport

### 7.1 Monoaminergic Balance

The brain uses monoaminergic neurotransmitters, which include serotonin and dopamine, to control both motivation levels and fatigue perception, as well as all neural activities. Central fatigue development, together with exercise tolerance, both depend on how these systems interact with each other. Dopamine drives motor activation and makes reward processing possible while helping people make decisions about how much effort to put into their tasks. People generally associate serotonin with mood control because it makes them feel sleepy and affects their ability to judge how hard they work (Yang, Feng, et al. 2024).

Central serotonin levels increase during intense exercise, while dopaminergic activity experiences a decline. The shift in serotonin–dopamine balance leads to decreased motivation and stronger effort perception, which results in lower central motor command. The increased serotonergic control leads to decreased motor neuron activity, which causes both lower motor unit activation and faster performance decrease. Athletes experience central fatigue because this neurochemical pattern causes them to feel exhausted while their peripheral muscle strength remains intact (Pitts and Bhatt 2023; Koozehchian et al. 2025).

### 7.2 Psychological Resilience and Motivation

The ability to endure performance challenges that arise from training stress, competitive pressure, and environmental difficulties depends on two psychological factors. The hypothalamic–pituitary–adrenal (HPA) axis controls the body's physical response to stress through its primary function of cortisol release. Humans need initial cortisol increments to support energy release, plus body modifications. However, continuous cortisol elevation causes cognitive decline, mood disorders, sleep interruptions, and increased fatigue perception (Wu et al. 2024).

The HPA axis dysregulation results in two types of mental fatigue through neurotransmitter disruption, prefrontal cortex damage, and heightened emotional and physical stress. Increased stress levels create a negative impact on attention control, which leads to poor decision‐making and faster development of exhaustion symptoms. The body fails to adapt to stress, which results in negative training outcomes that lead to overreaching and inconsistent performance during training sessions (Li and Pan 2025; Habay et al. 2023).

## 8 Central Governor Theory and Cognitive Control of Performance

The central governor theory explains how the brain controls physical performance to keep the body functioning normally and to stop major breakdowns from happening. The model shows that exercise performance depends on two factors, which include restrictions on the body and the brain's ability to control motor functions through incoming information, previous knowledge, and emotional states. The central nervous system uses metabolic information, thermal information, cardiovascular information, and perceived effort information to control the capacity of the body to perform exercise and its energy management system (Pessiglione et al. 2025).

The effort perception system of this framework creates effort perceptions through its central system, which processes both physiological signals and psychological signals from the body. The body uses perceived effort as a regulatory signal that determines how much effort someone will put into their current exercise work. The brain controls motor unit recruitment and pacing to create performance limits, which protect the body from reaching dangerous energy levels. Athletes use central regulation to decrease their workout intensity because they want to stop exercising before they reach complete muscular exhaustion (Foster et al. 2023; Roth et al. 2025; Grivas 2025; Yuan et al. 2023).

## 9 Neurotransmission and Motor Output

Acetylcholine (ACh) serves as the main neurotransmitter that enables signal transmission through the neuromuscular junction (NMJ) to initiate skeletal muscle contraction from neural impulses. The motor neuron releases ACh, which binds to nicotinic receptors found on muscle fibers. This binding leads to muscle fiber depolarization, which results in muscle fiber activation. Proper cholinergic function serves as the necessary foundation that supports muscle strength development, reaction time improvement, and the ability to maintain muscle contraction. The nootropic compounds choline, alpha‐GPC, and citicoline enhance ACh synthesis and availability, which results in improved neuromuscular performance and motor learning and resistance to neuromuscular fatigue during extended workouts or intense exercise (Khairullin et al. 2023).

Dopamine functions as a key neurotransmitter underlying motivation, reward processing, motor control, and exercise effort perception. People who experience increased dopamine activity demonstrate stronger motivation, decreased fatigue perception, and a better ability to handle physical challenges (Gharakhanlou and Fasihi 2023). Dopamine helps athletes in endurance sports by maintaining their performance level and postponing their central fatigue because it affects both their mood and their ability to control their body movements. People who use nootropic and ergogenic supplements such as L‐tyrosine, caffeine, and *Rhodiola rosea* will experience better mental resilience, motivation, and endurance performance because these substances help with dopamine production and signaling (Malík and Tlustoš 2023; Hayman et al. 2025).

## 10 Brain–Muscle Energy Coupling Mechanisms

### 10.1 Creatine–Phosphate Shuttle

**FIGURE 5:**
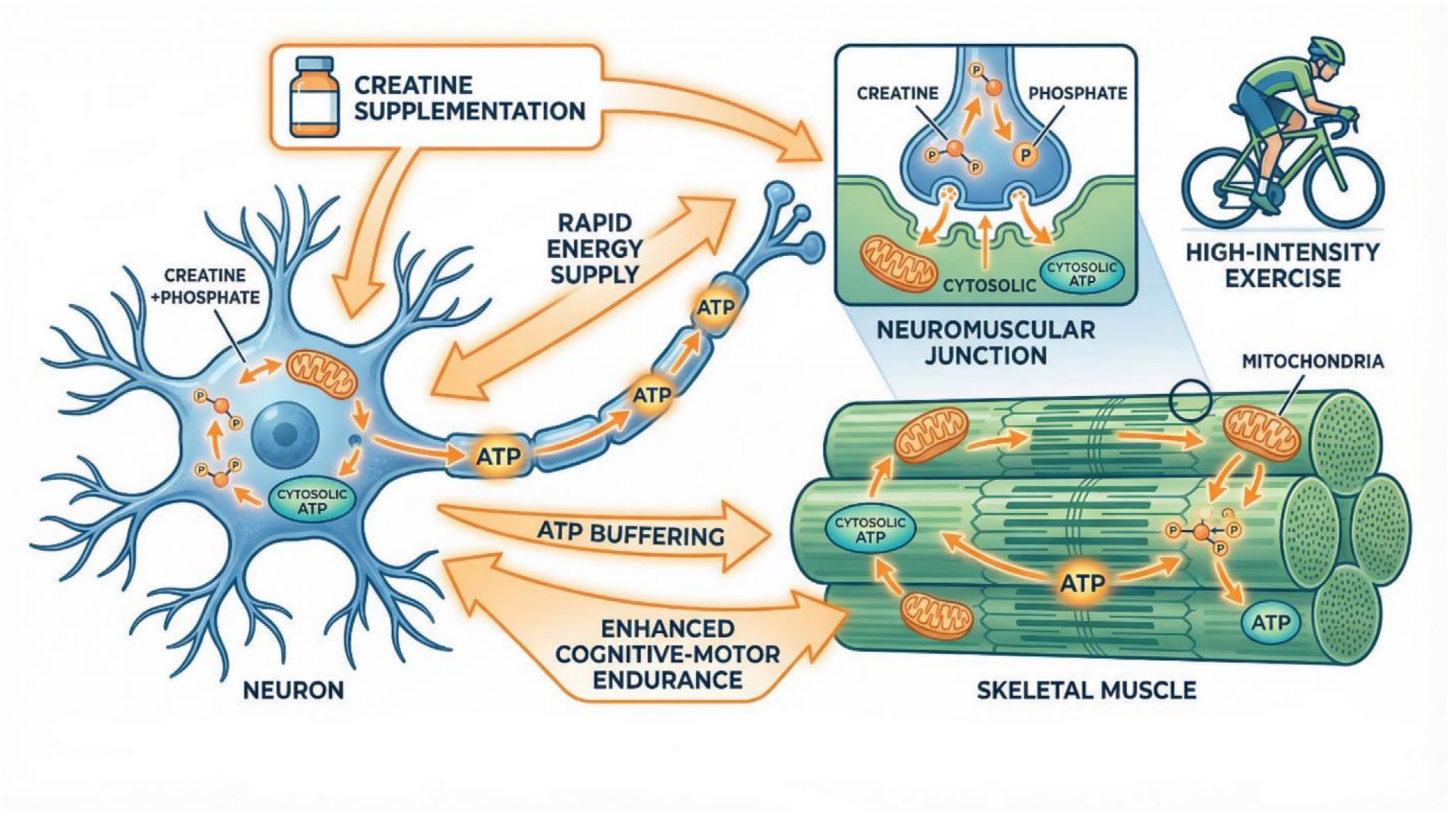
Brain–muscle energy coupling via creatine–phosphate shuttle.

The creatine–phosphate (phosphocreatine) shuttle serves as an essential system that generates ATP at high speed for tissues that need power during changing energy requirements (Candow et al. 2026). Creatine kinase enables the reversible movement of a phosphate group from phosphocreatine to ADP, which produces ATP at high speed during times of intense neural activity and muscle contraction. The system enables neurons to support synaptic transmission, ion pump operation, and action potential propagation while muscle cells use it to maintain ATP levels during short‐lasting high‐intensity exercise (Su 2025). Creatine supplementation has been shown to increase intramuscular and cerebral phosphocreatine stores, which leads to improved power output, fatigue resistance, and cognitive function during metabolic stress. The dual function of creatine enables it to function as a fundamental energy regulator that affects both brain activity and muscle energy systems during athletic performance (Chen et al. 2023) (Figure 5).

### 10.2 Neurovascular Coupling

**FIGURE 6:**
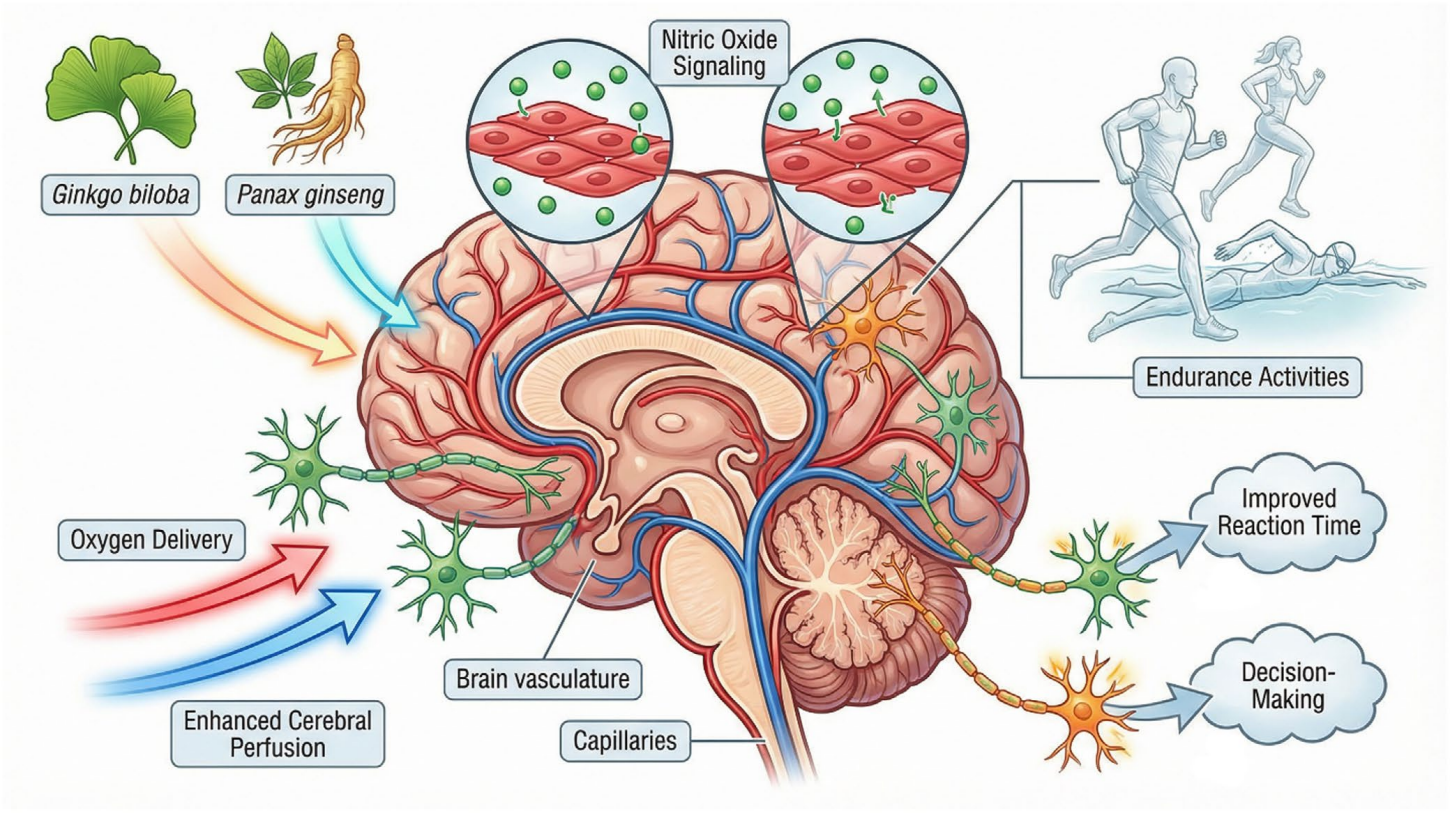
Neurovascular coupling and cerebral perfusion.

The physiological process that handles the delivery of oxygen and nitric oxide through cerebral blood flow functions in response to neuronal activity increases, which directly produce higher levels of local cerebral blood flow. The mechanism of this system functions to ensure that oxygen and glucose reach all active brain areas. The body uses nitric oxide to control blood vessel expansion, which manages blood circulation to the brain and the rest of the body (Yang, Zhao, et al. 2024). The brain uses neurovascular coupling during physical activity to maintain motor cortex function, reaction time, and executive control while delivering oxygen to active muscles. Nootropic and performance supplements such as L‐arginine, L‐citrulline, dietary nitrates, and polyphenols may enhance NO bioavailability and endothelial function, which leads to better cerebral blood flow, cognitive endurance, and exercise performance (Zhong et al. 2025) (Figure 6).

## 11 Molecular and Biochemical Mechanisms Underlying Nootropic Action

### 11.1 Neuroendocrine and Metabolic Regulation

Nootropics and adaptogens like *Rhodiola rosea* and Ashwagandha, together with selected amino acid supplements, activate the hypothalamic–pituitary–adrenal (HPA) axis system. The system helps athletes maintain proper catecholamine levels because it decreases cortisol production, which happens during physical or mental stress. The body requires lower cortisol levels because they stop skeletal muscle catabolic pathways while protecting brain function from chronic stress, which helps people stay mentally strong during intense exercise and extended physical activity (Malík and Tlustoš 2023).

Mitochondrial biogenesis, together with energy efficiency, functions as the fundamental mechanism that enables people to maintain their cognitive capabilities and physical performance (Voronina 2023).

### 11.2 Oxidative Stress Modulation and Neuroprotection

Exercise‐induced oxidative stress impairs neuronal function because it decreases synaptic efficiency and triggers central and peripheral fatigue. Many natural nootropics demonstrate strong antioxidant effects, which protect against reactive oxygen species (ROS) while increasing the body's natural antioxidant defense systems, which include superoxide dismutase and glutathione peroxidase. The effects of these substances provide protection to neurons and glial cells against oxidative damage while they keep mitochondrial function intact and enable proper neurotransmission during times of intense cognitive and physical activity (Hannan et al. 2022).

### 11.3 BDNF and Synaptic Plasticity

Brain‐derived neurotrophic factor (BDNF) serves as the primary connector that links synaptic plasticity and learning and memory consolidation. Nootropic supplements increase BDNF expression, which leads to better neuroplasticity that helps people learn motor skills and cognitive skills. BDNF signaling increases dendritic growth and synapse formation and long‐term potentiation, which together improve executive function and motor coordination. These adaptations apply most to sports that need fast learning of difficult skills, flexible decision‐making, and ongoing mental focus (Schifano et al. 2025).

### 11.4 Nitric Oxide Signaling and Cerebral Perfusion

Nitric oxide (NO) serves as a primary vasodilator that controls both internal body systems and brain blood circulation, together with oxygen transport and metabolic interactions between brain tissue and muscles. Nootropic and nutraceutical interventions, including L‐arginine, L‐citrulline, and polyphenol‐rich compounds, enhance NO bioavailability, which results in better endothelial function and neurovascular coupling. Increased blood flow to the brain provides sufficient oxygen and nutrients to active brain areas, which enables people to maintain focus and respond quickly while they perform vigorous exercise for extended periods. Improved skeletal muscle blood flow through NO results in better aerobic performance, which helps to achieve quicker recovery times while reducing peripheral exhaustion (Fong 2024).

## 12 Mechanistic Evaluation of Major Nootropic Supplements

### 12.1 Vitamins

B‐vitamins (B6, B12, folate) serve as essential cofactors that enable the production of neurotransmitters that include dopamine, serotonin, and acetylcholine. B‐vitamin levels, which reach optimal levels, enable individuals to process thoughts and sustain their focus while performing motor activities. Vitamin D controls the production of neurotrophic factors such as BDNF while it also controls calcium‐dependent neurotransmission, which assists in maintaining proper neuromuscular function and muscle strength. Antioxidant vitamins (C and E) decrease oxidative damage to neurons and skeletal muscle, which enables them to protect against fatigue‐related injuries and sustain cognitive and physical capabilities throughout extended physical activity (Jędrejko et al. 2023).

### 12.2 Amino Acids

Nootropic substances, which contain amino acids, control how neurotransmitters spread in the body while they also affect how neurons respond to external stimuli. The body uses tyrosine to produce catecholamines, which include dopamine and noradrenaline, so it helps people become more motivated and alert while they work through their cognitive and physical challenges. Theanine helps people achieve relaxation without becoming sleepy because it controls neurotransmitter activity, which helps them focus better. Taurine functions as both a neuromodulator and an antioxidant because it protects brain cells and boosts their ability to send signals. Tryptophan serves as a serotonin precursor, which helps regulate mood and fatigue during extended endurance training (Jędrejko et al. 2023).

### 12.3 Creatine

Creatine boosts synaptic transmission and neurotransmitter recycling in neurons, which enhances attention, memory, and reaction time. The muscles utilize creatine to increase their force output and their ability to operate with neuromuscular efficiency while extending their endurance to fatigue. The dual action of creatine on brain function and muscle performance creates special value for sports that need athletes to perform brief periods of maximum effort while they continue to think.

### 12.4 Noopept

Noopept operates as a nootropic that scientists developed from synthetic peptides to enhance neuroplasticity by increasing BDNF levels and modulating NMDA receptor functions. The process improves long‐term potentiation together with synaptic development while memory retention tracks advanced motor learning and strategic decision‐making. Noopept provides users with mild neuroprotective effects and antioxidant properties, which help them maintain their cognitive abilities during extended mental challenges and intense physical workouts.

## 13 Clinical Evidence and Human Validation in Athletic Populations

The research shows evidence which proves that military personnel have physiological and cognitive requirements that match those of elite athletes. Barringer et al. (2018) assessed a multi‐ingredient nootropic supplement in active‐duty US soldiers and reported improvements in mood state, stress resilience, and marksmanship performance. The research shows that nootropics benefit athletes who compete in sports which require them to think clearly under extreme pressure.

The specific botanical nootropics show positive potential for their use. Babault et al. (2021) reported that Salvia supplementation improved cognitive performance during intense cycling exercise because it protected users from mental fatigue that occurs with exercise. The researchers Koozehchian et al. (2025) demonstrated that *Rhodiola rosea* supplementation improved both cognitive and anaerobic performance in resistance‐trained athletes, which showed a direct connection between dosage and its impacts.

People now prefer products without caffeine because they think that using stimulant products will lead to dependence issues. The study by Tartar et al. (2021) and his research team established that a non‐stimulant energy supplement produced greater cognitive alertness and specific physical advantages without showing any heart‐related side effects. The study conducted by Razazan et al. (2025) showed that elite wrestlers performed better cognitively after they consumed both caffeine and L‐theanine because this combination prevented them from experiencing excessive excitement. The accomplishment of required results depends on safety elements which need assessment through regulatory processes. Jędrejko et al. (2023) discovered through their research that commercial nootropics contained unauthorized substances which could potentially serve as doping agents. The study conducted by Pastina and Stewart (2025) found that active individuals used more nootropics because they believed these substances would improve cognitive abilities and training performance, which demonstrates the necessity for doctors to supervise their consumption.

## 14 Future Perspectives

- Genetic variations that affect neurotransmitter pathways, metabolic processes, and neuroplasticity development determine how people react to nootropic substances.
- The method of designing supplements according to individual neurogenetic profiles will create the highest effectiveness while reducing all adverse reactions.
- The combination of specific cognitive and motor training exercises together with supplements will lead to enhanced performance through improved attention, reaction time, decision‐making abilities, and motor learning skills.
- The gut microbiota controls the metabolic process of neuroactive compounds, the production of neurotransmitters, and the body's response to inflammatory substances.
- The gut–brain axis presents an opportunity to improve nootropic drugs because it enhances their ability to be absorbed and their overall effectiveness in endurance sports and cognitively demanding activities.
- The system uses wearable sensors, which include EEG, heart rate variability, and cognitive workload sensors to deliver immediate information about mental exhaustion, central drive, and neuromuscular coordination.
- The system enables users to determine when to take nootropics and what amount to take for their best performance.

## 15 Conclusion

The use of nootropic supplements has proven to be an effective method for improving both cognitive functions and physical abilities in athletes through their impact on brain‐to‐muscle systems that operate together. The nootropic substances work by altering the major neurotransmitters dopamine, acetylcholine, serotonin, and GABA, which leads to changes in human motivation, focus, fatigue sensing, and body movement control. The body uses metabolic pathways together with molecular pathways, which include the creatine–phosphate shuttle and mitochondrial AMPK/PGC‐1α signaling and BDNF‐mediated synaptic plasticity and neurovascular coupling to provide energy and maintain efficient brain function and body movement. The advantages that exist for particular types of exercise demonstrate that endurance athletes receive benefits through improved central fatigue resistance and blood flow maintenance, while strength and power athletes experience better neuromuscular control and ATP energy storage, and solution athletes show better attention skills and their ability to learn motor skills and maintain control over their movements.

## Author Contributions

**Zheng Yi:** writing – original draft, supervision, project administration.

## Funding

The author has nothing to report.

## Conflicts of Interest

The author declares no conflicts of interest.

## Data Availability

The data that support the findings of this study are available from the corresponding author upon reasonable request.
